# Clinicopathological and Molecular Features of Patients with Early and Late Recurrence after Curative Surgery for Colorectal Cancer

**DOI:** 10.3390/cancers13081883

**Published:** 2021-04-14

**Authors:** Yuan-Tzu Lan, Shih-Ching Chang, Pei-Ching Lin, Chun-Chi Lin, Hung-Hsin Lin, Sheng-Chieh Huang, Chien-Hsing Lin, Wen-Yi Liang, Wei-Shone Chen, Jeng-Kai Jiang, Shung-Haur Yang, Jen-Kou Lin

**Affiliations:** 1 Division of Colon & Rectal Surgery, Department of Surgery, Taipei Veterans General Hospital, Taipei 11217, Taiwan; changsc@vghtpe.gov.tw (S.-C.C.); cclin15@vghtpe.gov.tw (C.-C.L.); hhlin7@vghtpe.gov.tw (H.-H.L.); schuang5@vghtpe.gov.tw (S.-C.H.); wschen@vghtpe.gov.tw (W.-S.C.); jkjiang@vghtpe.gov.tw (J.-K.J.); yangsh@vghtpe.gov.tw (S.-H.Y.); jklin@vghtpe.gov.tw (J.-K.L.); 2 Department of Surgery, Faculty of Medicine, School of Medicine, National Yang Ming Chiao Tung University, Taipei 112, Taiwan; 3 Department of Clinical Pathology, Yang-Ming Branch, Taipei City Hospital, Taipei 11146, Taiwan; b7901127@tmu.edu.tw; 4 Department of Health and Welfare, University of Taipei, Taipei 11153, Taiwan; 5 Division of Genomic Medicine, National Health Research Institutes, Zhunan 35053, Taiwan; jameslin@fcbiotech.com.tw; 6 Department of Pathology, Taipei Veterans General Hospital, Taipei 11217, Taiwan; wyliang@vghtpe.gov.tw; 7 Department of Surgery, National Yang Ming Chiao Tung University Hospital, Yilan 26058, Taiwan

**Keywords:** colorectal cancer, early recurrence, late recurrence, prognostic factor, genetic alteration

## Abstract

**Simple Summary:**

Colorectal cancer patients with early recurrence had advanced pathological node categories, pathological tumor, node, metastasis stages, adjuvant chemotherapy treatment, a worse overall survival rate, more liver metastases and more *APC* mutations than those with late recurrence. Patients with right-sided colon cancer tended to have early recurrence than those with left-sided colon cancer or rectal cancer. The postrecurrence survival rates were not significantly different between patients with early and late recurrence, which was observed in right-sided colon, left-sided colon and rectum. Multivariate analysis showed that old age, early recurrence, multiple-site recurrence and *BRAF* and *NRAS* mutations were independent prognostic factors.

**Abstract:**

Background: Few reports have investigated genetic alterations between patients with early and late recurrence following curative surgery for colorectal cancer (CRC). Methods: A total of 1227 stage I–III CRC patients who underwent curative resection were included retrospectively. Among them, 236 patients had tumor recurrence: 139 had early (<2 years after surgery) and 97 had late (≥2 years after surgery) recurrence. Clinicopathological features and genetic alterations were compared between the two groups. Results: Compared to those with late recurrence, patients with early recurrence were more likely to have advanced pathological node (N) categories; tumor, node, metastasis (TNM) stages; adjuvant chemotherapy treatment; liver metastases; *APC* mutations; and worse five-year overall survival rates. Patients with right-sided colon cancer were more likely to develop early recurrence than were those with left-sided colon cancer or rectal cancer. Regarding rectal cancer, patients with early recurrence were more likely to be at advanced pathological N categories and TNM stages than those with late recurrence. Multivariate analysis revealed old age, early recurrence, multiple-site recurrence, and *BRAF* and *NRAS* mutations to be independent prognostic factors. Conclusion: CRC patients with early recurrence have a worse OS rate and more *APC* mutations than those with late recurrence.

## 1. Introduction

Approximately 1.8 million new cases of colorectal cancer (CRC) are diagnosed annually, making it the third most commonly diagnosed malignancy and the second-leading cause of cancer deaths worldwide [1]. In Taiwan, colorectal cancer (CRC) is the most common cancer type and the third leading cause of cancer-related death [2]. As CRC accounts for approximately 15,000 new cancer diagnoses and 5700 cancer-related deaths every year, it is a major public health problem. It has been reported that the recurrence rate following curative surgery for CRC is 26–31%, with recurrence within five years after surgery in most cases [3,4].

The definition of the time period for early recurrence varies among studies, ranging from one to three years after surgery [5,6,7,8]. Although it has been reported that CRC patients with early recurrence have worse survival than those with late recurrence, no significant difference in the clinicopathological features was observed between those with early and late recurrence [6]. Nonetheless, *KRAS* mutation is more common in CRC patients with early tumor recurrence [6]. In our previous study [7], patients with multiple organ recurrence tended to die within two years after recurrence. However, differences in genetic alterations between CRC patients with single-site recurrence and those with multiple-site recurrence remain unclear.

To date, there have been few reports regarding the correlation between mutational profiles, recurrence pattern, and prognosis of CRC patients with early and late recurrence. Hence, we sought to determine whether the molecular profiles of patients with early and late recurrence differ, which might explain the difference in prognosis between these two groups. The aim of the present study was to compare differences in clinicopathological and mutational profiles between CRC patients with early and late recurrence.

## 2. Materials and Methods

### 2.1. Patient Enrollment

Between 2000 and 2010, a total of 1227 stage I–III CRC patients who underwent curative resection and for whom tumor samples stored in the biobank of Taipei Veterans General Hospital were available were included in this retrospective study. Among them, 236 patients (19.5%) had tumor recurrence following surgery: Of these, 139 had early recurrence and 97 had late recurrence. Early recurrence was defined as tumor recurrence diagnosed within two years after surgery; late recurrence was defined as tumor recurrence diagnosed two years or more after surgery. Clinicopathological features and genetic alterations were compared between the two groups. Written informed consent for sample collection and storage in the biobank was provided by all enrolled patients. The present study was approved by the Institutional Review Board of Taipei Veterans General Hospital.

Patients who received preoperative chemoradiotherapy or palliative surgery, who did not receive surgical treatment for primary CRC, who did not have available tumor tissue in the biobank, who underwent emergent operations, or who died within 30 days after surgery were excluded. Right-sided colon cancer was defined as a tumor located from the cecum to the transverse colon; left-sided colon cancer was defined as a tumor extending from the splenic flexure to the sigmoid colon.

After surgery, patients were followed up every three months for the first two years and every six months thereafter. In addition, carcinoembryonic antigen analysis, abdominal sonogram, chest radiography, and abdominal computerized tomography were arranged if needed. Proton emission tomography or magnetic resonance imaging was performed when the carcinoembryonic antigen level was elevated without confirmation of the site of tumor recurrence. Tumor recurrence was evaluated according to the following variables: timing (early/<2 years and late/>2 years), site (local, liver, lung, peritoneum, bone, and other sites) and number of sites (single site or multiple sites). Patients with resectable synchronous or metachronous metastasis were treated with surgery and adjuvant chemotherapy based on folinic acid, fluorouracil, and oxaliplatin (FOLFOX). Targeted therapies such as bevacizumab, cetuximab, and panitumumab were not reimbursed by the Taiwan National Health Insurance Administration before 2010.

### 2.2. DNA Extraction and Mutational Analysis of a 12-Gene Panel

DNA extraction was performed using a QIAamp DNA Tissue Kit (Qiagen, Valencia, CA, USA). A 12-gene panel for the identification of 139 mutations selected from among hotspots was assessed according to the Catalog Of Somatic Mutations In Cancer (COSMIC) database and previous studies [9,10]. As described in a previous report [11], the MassArray method was used to detect 139 hotspot mutations in 12 genes.

### 2.3. Microsatellite Instability (MSI) Analysis

Five microsatellite markers; namely, D5S345, D2S123, BAT25, BAT26, and D17S250, were used to define the MSI phenotype according to international criteria [12]. MSI-high tumors were defined as those with two or more positive MSI markers; microsatellite stable (MSS) tumors were defined as those with zero or one positive MSI marker.

Mutational analysis of a 12-gene panel and MSI analysis were performed on tissue specimens of primary CRC stored in the biobank of Taipei Veterans General Hospital. Biopsy tissues of recurrent tumors were not analyzed for mutational and MSI profiles. The investigators were unaware of the clinical outcome of the patients when analyzing the genetic mutations and MSI phenotype.

### 2.4. Statistical Analysis

IBM SPSS Statistics 25.0 (IBM Corp., Armonk, NY, USA) was used to perform the statistical analyses. Clinicopathological features were compared using chi-squared and two-tailed Fisher’s exact tests. Numerical values were compared using Student’s *t*-test. Overall survival (OS) was considered as the time from the date of diagnosis to the date of death. Kaplan–Meier survival curves were compared using the log-rank test. Univariate and multivariate Cox regression analyses were employed to assess the impact of molecular and clinicopathological features on OS. Variables differing significantly in univariate analysis were included in multivariate analysis based on logistic regression. Tumor recurrence was the primary endpoint, and OS was the secondary endpoint. A *p* value less than 0.05 was defined as statistically significant.

## 3. Results

### 3.1. Clinicopathological Features and Genetic Alterations

Among the 236 CRC patients who experienced tumor recurrence after curative surgery, 139 (58.9%) with recurrence within two years were considered the early recurrence group, whereas 97 patients with recurrence ≥2 years following surgery were considered the late-recurrence group. The follow-up time was 48.4 ± 46.0 months and 80.0 ± 45.5 months in the early- and late-recurrence groups, respectively. In the early-recurrence group, the median recurrence time was 12.5 months and the median overall survival time was 32.1 months. In the late-recurrence group, the median recurrence time was 40.4 months and the median overall survival time was 65.7 months. All variables reported in the first column of Table 1 and Table 2 refer to primary colorectal cancer. As shown in Table 1, compared to patients with late recurrence, those with early recurrence were more likely to be at advanced pathological N categories and TNM stages, to have undergone adjuvant chemotherapy, and to carry *APC* mutations. Additionally, patients with right-sided colon cancer were more likely to develop early recurrence than those with left-sided colon cancer or rectal cancer. Because the high variance inflation factor was more than 1.5, we excluded the pathologic N category in multiple testing correction logistic regression analysis. Ultimately, four covariates (tumor location, adjuvant chemotherapy, pathological TNM stage, and *APC* mutations) were included in this analysis, which demonstrated that patients with early recurrence were more likely to have right-sided colon cancer, be at advanced pathological TNM stages, and harbor *APC* mutations than patients with late recurrence. The Cramar value is provided in Table 1. The variance inflation factor was 1.02 and the Bayesian information criterion was −968.836.

Regarding right-sided colon cancer, there was no significant difference in clinicopathological features and genetic mutations between patients with early and late recurrence, as indicated in Table 2. For left-sided colon cancer, however, more patients with early recurrence had *PIK3CA* and *NRAS* mutations than patients with late recurrence. For rectal cancer, patients with early recurrence tended to have advanced pathological N categories and TNM stage and to carry *APC* mutations compared with patients with late recurrence.

**Figure 1:**
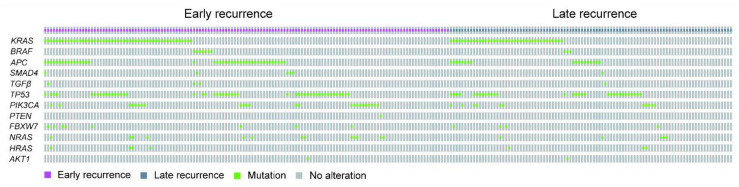
The oncoprint of genetic mutations for all assessed colorectal cancer (CRC) patients with early and late recurrence.

**Figure 2:**
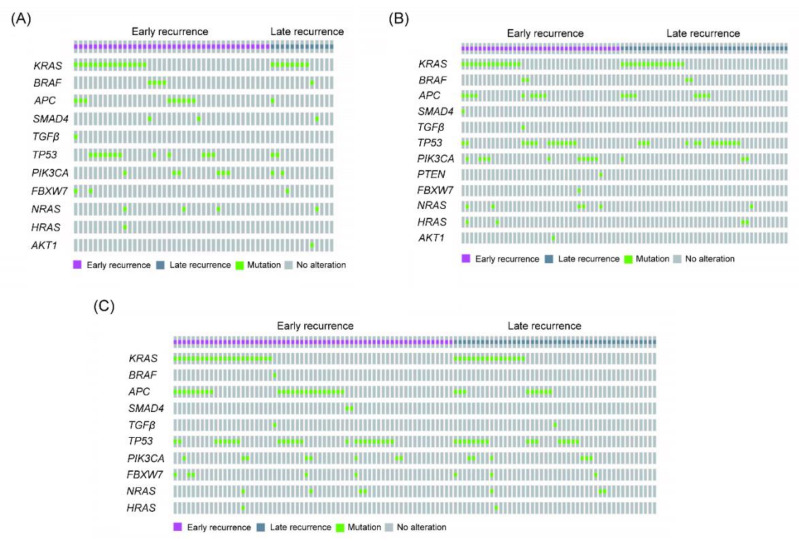
The oncoprint of genetic alterations for patients with early and late recurrence of right-sided and left-sided CRC. The mutation profiles are shown as follows: (**A**) right-sided colon cancer, (**B**) left-sided colon cancer, and (**C**) rectal cancer.

As illustrated in Figure 1 and Table 1, the gene most commonly mutated in patients with early or late recurrence was *KRAS*, followed by *TP53*, *APC*, and *PIK3CA*. As above, the most commonly mutated gene in those with right-sided colon cancer and early recurrence was *KRAS*, followed by *TP53*, *APC,* and *PIK3CA* (Figure 2A and Table 2), though the most commonly mutated gene in those with late recurrence was *KRAS*, followed by *TP53,* and *PIK3CA*. In left-sided colon cancer, the most commonly mutated gene in those with early recurrence was *KRAS*, followed by *TP53*, *PI3KCA*, and *APC*, and that in those with late recurrence was *KRAS*, followed by *TP53*, *APC,* and *PIK3CA* (Figure 2B and Table 2). In rectal cancer, the most commonly mutated genes in patients with early recurrence were *TP53* and *APC*, followed by *KRAS* and *PIK3CA* (Figure 2C and Table 3). In brief, the four most commonly mutated genes were the same in those with early and late recurrence.

As indicated in Table 3, multiple-site recurrence among those with early recurrence was associated with a higher rate of *NRAS* mutations than single-site recurrence. Conversely, there was no difference in genetic mutations between those with single-site recurrence and those with multiple-site recurrence among patients with late recurrence.

### 3.2. Recurrence Patterns

As shown in Table 4, patients with early recurrence had more liver metastases (44.6% vs. 33.0%, *p* = 0.048) and fewer metastases in other organs (other than local and the liver, lung, peritoneum, and bone) than those with late recurrence (4.3% vs. 15.5%, *p* = 0.003). For right-sided and left-sided colon cancer, there was no significant difference in the recurrence pattern between patients with early and late recurrence. With regard to rectal cancer, patients with late recurrence were more likely to have recurrence in other organs (other than local and the liver, lung, peritoneum, and bone) and multiple-site recurrence (33.3% vs. 12.9%, *p* = 0.011) than patients with early recurrence.

### 3.3. Survival Analysis

**Figure 3:**
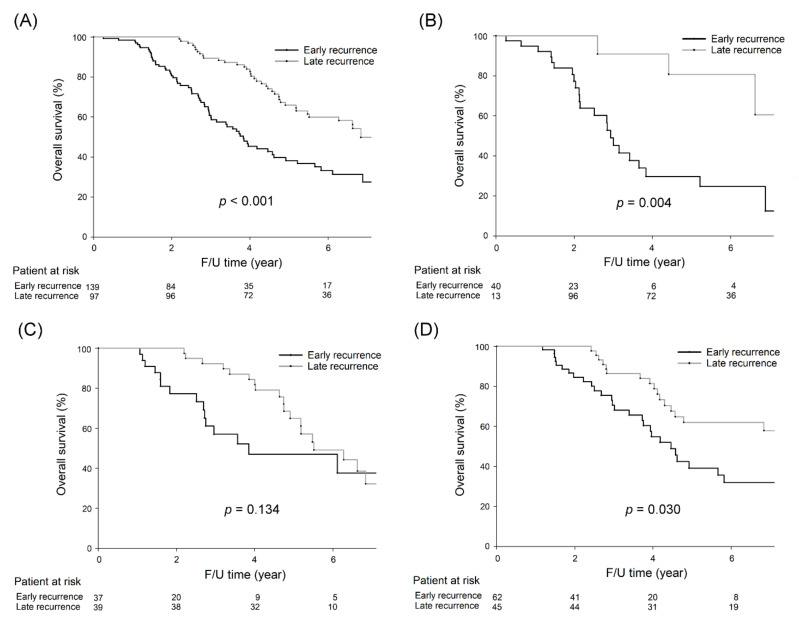
Five-year OS rates were significantly lower in CRC patients with early recurrence than in those with late recurrence (38.2% vs. 65.9%, *p* < 0.001). For right-sided colon cancer, five-year OS rates were significantly lower in CRC patients with early recurrence than in those with late recurrence (29.7% vs. 80.8%, *p* = 0.004). For left-sided colon cancer, five-year OS rates were not significantly different between CRC patients with early recurrence and those with late recurrence (47.0% vs. 64.9%, *p* = 0.134). For rectal cancer, five-year OS rates were significantly lower in CRC patients with early recurrence than in those with late recurrence (39.1% vs. 62.0%, *p* = 0.030). The survival curves are shown as follows: (**A**) all CRC patients, (**B**) right-sided colon cancer patients, (**C**) left-sided colon cancer patients, and (**D**) rectal cancer patients.

As depicted in Figure 3A, patients with early recurrence had a worse five-year OS rate than those with late recurrence (38.2% vs. 65.9%, *p* < 0.001). For right-sided colon cancer, those with early recurrence had a worse five-year OS rate than those with late recurrence (29.7% vs. 80.8%, *p* = 0.004, Figure 3B), although there was no significant difference in the five-year OS rate between patients with early and late recurrence for left-sided colon cancer (47.0% vs. 64.9%, *p* = 0.134, Figure 3C). Regarding rectal cancer, patients with early recurrence had a significantly worse five-year OS rate than those with late recurrence (39.1% vs. 62.0%, *p* = 0.030, Figure 3D).

As shown in Table 5, univariate analysis results revealed five covariates to be significantly correlated with OS: age, pathological N category, early recurrence, and *BRAF* and *NRAS* mutations. The five covariates were included in multivariate analysis, with age, early recurrence, and *BRAF* and *NRAS* mutations remaining independent prognostic factors affecting OS.

The univariate analysis results shown in Table 6 demonstrate that three covariates correlated significantly with survival: age and *BRAF* and *NRAS* mutations. These three covariates were included in multivariate analysis, which revealed age and *NRAS* mutations to be associated with survival. The Cramer value is shown in Table 6. The variance inflation factor was 1.0, and the Bayesian information criterion was −961.281.

**Figure 4:**
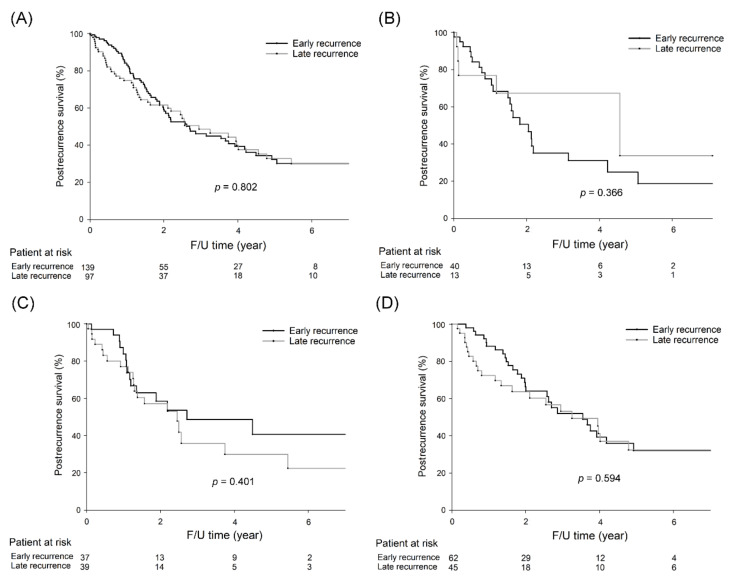
Five-year postrecurrence survival rates were not significantly different between CRC patients with early recurrence and those with late recurrence (32.4% vs. 32.8%, *p* = 0.802). For right-sided colon cancer, five-year postrecurrence rates were not significantly different between patients with early recurrence and patients with late recurrence (24.9% vs. 33.7%, *p* = 0.366). For left-sided colon cancer, five-year postrecurrence rates were not significantly different between patients with early recurrence and patients with late recurrence (40.6% vs. 29.9%, *p* = 0.401). For rectal cancer, five-year postrecurrence rates were not significantly different between patients with early recurrence and patients with late recurrence (32.0% vs. 32.4%, *p* = 0.594). The survival curves are shown as follows: (**A**) all CRC patients, (**B**) right-sided colon cancer patients, (**C**) left-sided colon cancer patients, and (**D**) rectal cancer patients.

Overall, five-year postrecurrence survival rates were not significantly different between CRC patients with early recurrence and those with late recurrence (Figure 4A; 32.4% vs. 32.8%, *p* = 0.802). Furthermore, the five-year postrecurrence survival rates did not differ significantly among patients with early recurrence and those with late recurrence of right-sided colon cancer (24.9% vs. 33.7%, *p* = 0.366, Figure 4B), left-sided colon cancer (40.6% vs. 29.9%, *p* = 0.401, Figure 4C), and rectal cancer (32.0% vs. 32.4%, *p* = 0.594, Figure 4D).

## 4. Discussion

To the best of our knowledge, the present study includes the largest population investigated for genetic alterations between CRC patients with early and late recurrence. The novel finding of the present study is that *APC* mutations were more common in CRC patients with early recurrence than in those with late recurrence, especially among those with rectal cancer. For patients with early recurrence, multiple-site recurrence was associated with a higher rate of *NRAS* mutations than single-site recurrence. Compared to late recurrence, early recurrence was an independent prognostic factor in CRC and was associated with recurrence, as well as with a poorer prognosis.

The major difference between the present study and others is that our results demonstrated that more CRC patients with early recurrence carried *APC* mutations than those with late recurrence, though other studies have shown that early recurrence was associated with more *KRAS* mutations than late recurrence. In the present study, the *KRAS* gene was among the top mutated genes in patients with both early and late recurrence, regardless of tumor location. It seems that *KRAS* mutations play an important role in CRC recurrence. Mutations in *APC*, *KRAS,* and *TP53* were detected in the cell-free DNA of CRC patients, indicating that these genes may serve as a tool for the early detection of tumor recurrence [13]. In addition, the top four mutated genes in our cohort of CRC patients with tumor recurrence included *KRAS*, *TP53*, *APC,* and *PIK3CA*, and mutations in these genes were observed in those with both early and late recurrence. Based on our results, physicians should be aware of tumor recurrence in patients with mutations in the above genes; for patients with *APC* mutations, surveillance for tumor recurrence is necessary, especially in the first few years after surgery.

Our results showed that patients with right-sided colon cancer were more likely to develop early recurrence than were those with left-sided colon cancer or rectal cancer, which was similar to the study of Eisenberg et al. [14]. In addition, patients with early recurrence were more likely to develop liver metastasis. We further investigated the correlation between *APC* mutations and recurrence patterns in our cohort of patients with early recurrence, and found that those with early recurrence and *APC* mutations were more likely to develop lung metastasis than those without *APC* mutations (50.0% vs. 29.9%, *p* = 0.023). A higher risk to liver metastasis has been reported for CRC with *APC* mutations, and *APC* mutations are frequently detected in lung metastasis tissues [15,16]. It seems that *APC* mutations play an important role in early recurrence and hematogenous metastasis in CRC. Nevertheless, when a recurrence involves the liver within two years of surgery, it is possible that those liver metastases are microscopic metastases not detected at the time of surgery. Overall, *APC* mutations might be associated with silent liver metastases rather than with early recurrence.

The association of *APC* mutations and a hereditary cancer syndrome of familial adenomatous polyposis (FAP) is well known. *APC* is a tumor-suppressor gene that plays a key role in the earliest step of CRC carcinogenesis. It encodes the APC protein, which is the main component of the β-catenin destruction complex involved in suppression of the Wnt/β-catenin pathway. Loss of APC function prevents the formation of this complex, resulting in the accumulation of β-catenin in the cytoplasm, which is later translocated to the nucleus, where it binds to TCF/LEF transcription factors. A study using CRC organoids enriched with cancer stem cells showed that noncanonical Hedgehog signaling is a positive regulator of the Wnt pathway and is required for the survival of colon cancer stem cells [17]. In the present study, among 236 CRC patients with tumor recurrence, the frequency of *APC* mutations was 37.5% and 24.5% in those with and without hereditary colorectal cancer, respectively (*p* = 0.251). Among the 220 patients without hereditary colorectal cancer, those with early recurrence more often had *APC* mutations than those with late recurrence (29.7% vs. 17.4%, *p* = 0.037). However, there was no significant difference in the frequency of *APC* mutations between patients with early recurrence and those with late recurrence among the 16 patients with hereditary colorectal cancer (36.4% vs. 40.0%, *p* = 0.889). In general, *APC* mutations appear to be associated with early tumor recurrence in patients without hereditary colorectal cancer. The detailed mechanism is unclear, and further in vivo and in vitro studies are required to validate our findings.

*APC*, a tumor-suppressor gene located on chromosome 5q21-q22, is involved in CRC carcinogenesis, and Wnt/β-catenin signaling is affected by *APC* mutation [18]. For example, an *APC*-knockout CRC mouse model revealed that restoration of *APC* function promoted cell differentiation and sustained tumor regression [19]. Nonetheless, the toxicity of Wnt pathway inhibitors to normal intestinal epithelium limits their clinical application [20]. The small molecule, truncated *APC* selective inhibitor (TASIN-1), can kill CRC cells with *APC* truncations while sparing wild-type *APC* cells [21]. In addition, in vivo studies have demonstrated that TASIN-1 can inhibit the growth of *APC*-truncated CRC cells without deleterious effects on the normal colonic epithelium, although it is not currently under clinical application.

Our results showed that *APC* mutations were involved in early recurrence but were not associated with OS, and that *BRAF* and *NRAS* are independent risk factors for reduced survival. The mechanisms of carcinogenesis related to these genes likely differ. In general, approximately 15–30% of CRCs arise from serrated lesions, and these lesions are characterized by genetic (*BRAF* or *KRAS* mutations) and epigenetic (CpG island methylator phenotype) alterations. Indeed, they are distinct from tumors arising from the conventional chromosomal instability pathway (CIN) pathway, and rarely present truncating *APC* mutations [22]. *BRAF* and *KRAS* mutations are considered drivers of the formation of serrated colorectal cancers [23]. Previous studies have also shown that *BRAF* and *NRAS* mutations are associated with shorter OS [24,25,26]. Based on our analysis, their prognostic roles are not related to the timing of recurrence.

Although our results demonstrated that among left-sided colon cancer patients, those with early recurrence were more likely to carry *PIK3CA* and *NRAS* mutations than those with late recurrence, the number of patients was small, and selection bias might have occurred. *BRAF* and *NRAS* mutations were found to be independent prognostic factors of poor OS in our cohort of CRC patients with tumor recurrence. It has been reported that *RAS* mutation and multiple-site recurrence are independent predictors of poor survival [27]. In addition, CRC patients with *BRAF* or *NRAS* mutation are resistant to anti-EGFR therapy [28]. For our cohort of CRC patients with early recurrence, those with multiple-site recurrence had more *NRAS* mutations than those with single-site recurrence, which has not yet been reported. Among those with left-sided colon cancer in our cohort, approximately 16.2% of the patients in our cohort with early recurrence harbored *NRAS* mutations. Consequently, anti-EGFR therapy may not be helpful for these subgroups of patients.

For CRC, few biomarkers have been introduced for treatment, including *RAS* and *BRAF* mutations, and MSI and CIMP status. Guinney et al. [29] classified CRC into four consensus molecular subtypes (CMSs): CMS1 tumors characterized by increased expression of genes associated with a diffuse immune infiltrate and features of MSI CRC; CMS2 tumors with strong upregulation of WNT and *MYC* downstream targets; CMS3 tumors with metabolic pathway dysregulation and *KRAS* mutations; and CMS4 tumors with clear upregulation of genes involved in the epithelial mesenchymal transition and associated with the activation of *TGFβ* signaling, angiogenesis, matrix-remodeling pathways, and the complement inflammatory system. However, these classifications do not predict drug response, limiting their clinical application. To identify biomarkers and predict drug sensitivity, the OncoTrack consortium developed preclinical models using a large biobank of CRC tumors, organoids, and xenografts [30]. By linking molecular profiles with drug-sensitivity patterns, a novel classification outperforming *RAS/RAF* mutations in predicting sensitivity to the EGFR inhibitor cetuximab was identified.

In the present study, multivariate analysis showed that old age, early recurrence, multiple-site recurrence, and *BRAF* and *NRAS* mutations were associated with poor prognosis. All these factors potentially influence prognosis. In addition to the traditional adenoma–carcinoma sequence and serrated pathway, the inflammatory pathway plays a major role in colorectal carcinogenesis [1]. In patients with inflammatory bowel disease (IBD), an estimated 2.4-fold higher risk of CRC (95% CI 2.1–2.7) compared with the general population has been reported for an average follow-up of 14 years [31]. The timing and frequency of molecular events in the inflammatory pathway are also distinct from the adenoma–carcinoma sequence, with *TP53* mutations as an early event and infrequent *APC* mutations late in carcinogenesis [32]. Due to the very low incidence of IBD and IBD-related CRC in Taiwan [33], data regarding IBD-related CRC in our study cohort were not available, and we could not elucidate the influence of the inflammatory pathway on tumor recurrence.

There are limitations to the present study. This is a retrospective study, and only patients with tumor tissue available in the biobank were enrolled; thus, selection bias exists. Despite the significant difference observed for some genetic mutations, bias may also exist due to the small sample size and low mutation rate. To validate our results, more patients from different countries and of different races need to be studied.

## 5. Conclusions

Our results showed that CRC patients with early recurrence had worse OS, more liver metastases, and more *APC* mutations than those with late recurrence. Postrecurrence survival rates were not significantly different between patients with early and late recurrence.

## Figures and Tables

**Table 1:** Clinicopathological features and mutation spectrum between early and late recurrence after curative surgery for colorectal cancer.

Variables	Univariate Analysis				Multiple Testing Correction Logistic Regression		
	Early Recurrence *n* = 139 *n* (%)	Late Recurrence *n* = 97 *n* (%)	Cramer Value	*p* Value	Odds Ratio	Confidence Interval	*p* Value
Age (years)			0.043	0.508			
<70	72 (51.8)	46 (47.4)					
≥70	67 (48.2)	51 (52.6)					
Sex			0.045	0.489			
Male	90 (64.7)	67 (69.1)					
Female	49 (35.3)	30 (30.9)					
Tumor location			0.199	**0.009**			**0.015**
Right-sided colon	40 (28.8)	13 (13.4)			1.000		
Left-sided colon	37 (26.6)	39 (40.2)			3.225	1.451–7.168	
Rectum	62 (44.6)	45 (46.4)			2.380	1.105–5.124	
Tumor differentiation			0.088	0.174			
Well to moderate	129 (92.8)	94 (96.9)					
Poor	10 (7.2)	3 (3.1)					
Lymphovascular invasion	37 (26.6)	19 (19.6)	0.081	0.212			
Adjuvant chemotherapy	94 (67.6)	48 (49.5)	0.182	**0.005**			
Pathological T category			0.088	0.605			
T1	1 (0.7)	1 (1.0)					
T2	11 (7.9)	9 (9.3)					
T3	106 (76.3)	78 (80.4)					
T4	21 (15.1)	9 (9.3)					
Pathological N category			0.223	**0.003**			**0.005**
N0	40 (28.8)	47 (48.5)			1.000		
N1	42 (30.2)	28 (28.9)			0.539	0.278–1.045	
N2	57 (41.0)	22 (22.7)			0.336	0.172–0.656	
Pathological TNM stage			0.201	**0.008**			
I	6 (4.3)	8 (8.2)					
II	34 (24.5)	39 (40.2)					
III	99 (71.2)	50 (51.5)					
MSI status			0.055	0.398			
MSS	129 (92.8)	87 (89.7)					
MSI-high	10 (7.2)	10 (10.3)					
Genetic mutations							
*TP53*	49 (35.3)	31 (32.0)	0.034	0.599			
*APC*	42 (30.2)	18 (18.6)	0.132	**0.043**	0.462	0.237–0.898	**0.023**
*PIK3CA*	24 (17.3)	11 (11.3)	0.082	0.208			
*BRAF*	7 (5.0)	3 (3.1)	0.047	0.466			
*KRAS*	51 (36.7)	39 (40.2)	0.036	0.584			
*NRAS*	13 (9.4)	5 (5.2)	0.078	0.232			
*HRAS*	4 (2.9)	3 (3.1)	0.006	0.924			
*FBXW7*	8 (5.8)	4 (4.1)	0.037	0.575			
*PTEN*	1 (0.7)	0	0.054	0.403			
*SMAD4*	5 (3.6)	1 (1.0)	0.218	0.218			
*TGFβ*	3 (2.2)	1 (1.0)	0.043	0.509			
*AKT1*	1 (0.7)	1 (1.0)	0.017	0.797			

MSI: microsatellite instability; MSS: microsatellite stable; TNM: tumor, node, metastasis; bold: statistically significant.

**Table 2:** Clinicopathological features between early and late recurrence of colorectal cancer according to tumor location.

Variables	Right-Sided Colon Cancer			Left-Sided Colon Cancer			Rectal Cancer		
	Early Recurrence *n* = 40 *n* (%)	Late Recurrence *n* = 13 *n* (%)	*p* Value	Early Recurrence *n* = 37 *n* (%)	Late Recurrence *n* = 39 *n* (%)	*p* Value	Early Recurrence *n* = 62 *n* (%)	Late Recurrence *n* = 45 *n* (%)	*p* Value
Age (years)			0.679			0.985			0.288
<70	18 (45.0)	5 (38.5)		20 (54.1)	21 (53.8)		34 (54.8)	20 (44.4)	
≥70	22 (55.0)	8 (61.5)		17 (45.9)	18 (46.2)		28 (45.2)	25 (55.6)	
Sex			0.942			0.354			0.722
Male	22 (55.0)	7 (53.8)		26 (70.3)	31 (79.5)		42 (67.7)	29 (64.4)	
Female	18 (45.0)	6 (46.2)		11 (29.7)	8 (20.5)		20 (32.3)	16 (35.6)	
Tumor differentiation			0.391			0.600			-
Well to moderate	33 (82.5)	12 (92.3)		34 (91.9)	37 (94.9)		62 (100)	45 (100)	
Poor	7 (17.5)	1 (7.7)		3 (8.1)	2 (5.1)		0	0	
Lymphovascular invasion			0.860			0.120			0.288
Absent	33 (82.5)	11 (84.6)		25 (67.6)	31 (79.5)		44 (71.0)	36 (80.0)	
Present	7 (17.5)	2 (15.4)		12 (32.4)	8 (20.5)		18 (29.0)	9 (20.0)	
Adjuvant chemotherapy			0.349			0.246			0.050
No	10 (25.0)	5 (38.5)		15 (40.5)	21 (53.8)		20 (32.3)	28 (51.1)	
Yes	30 (75.0)	8 (61.5)		22 (59.5)	18 (46.2)		42 (67.7)	22 (48.9)	
Pathological T category			0.747			0.452			0.678
T1	0	0		0	1 (2.6)		1 (1.6)	0	
T2	2 (5.0)	1 (7.7)		1 (2.7)	3 (7.7)		8 (12.9)	5 (11.1)	
T3	31 (77.5)	10 (76.9)		32 (86.5)	33 (84.6)		43 (69.4)	35 (77.8)	
T4	7 (17.5)	2 (15.4)		4 (10.8)	2 (5.1)		10 (16.1)	5 (11.1)	
Pathological N category			0.083			0.592			**0.020**
N0	10 (25.0)	5 (38.4)		12 (32.4)	17 (43.6)		18 (29.0)	25 (55.6)	
N1	11 (27.5)	6 (46.2)		14 (37.8)	13 (33.3)		17 (27.4)	9 (20.0)	
N2	19 (47.5)	2 (15.4)		11 (29.7)	9 (23.1)		27 (43.5)	11 (24.4)	
Pathological TNM stage			0.349			0.472			**0.018**
I	0	0		1 (2.7)	3 (7.7)		5 (8.1)	5 (11.1)	
II	10 (25.0)	5 (38.5)		11 (29.7)	14 (35.9)		13 (21.0)	20 (44.4)	
III	30 (75.0)	8 (61.5)		25 (67.6)	22 (56.4)		44 (71.0)	20 (44.4)	
MSI status			0.982			0.264			0.879
MSS	37 (92.5)	12 (92.3)		35 (94.6)	34 (87.2)		57 (91.9)	41 (91.1)	
MSI-high	3 (7.5)	1 (7.7)		2 (5.4)	5 (12.8)		5 (8.1)	4 (8.9)	

MSI: microsatellite instability; MSS: microsatellite stable; T: tumor; N: node; bold: statistically significant.

**Table 3:** The mutation spectrum of early and late recurrence in colorectal cancer stratified by number of recurrence site.

Gene	Early Recurrence			Late Recurrence		
	Single Site Recurrence *n* = 110 *n* (%)	Multiple Sites Recurrence *n* = 29 *n* (%)	*p* Value	Single Site Recurrence *n* = 73 *n* (%)	Multiple Sites Recurrence *n* = 24 *n* (%)	*p* Value
*TP53*	36 (32.7)	13 (44.8)	0.225	26 (35.6)	5 (20.8)	0.178
*APC*	34 (30.9)	8 (27.6)	0.729	12 (16.4)	6 (25.0)	0.349
*PIK3CA*	16 (14.5)	8 (27.6)	0.098	9 (12.3)	2 (8.3)	0.592
*BRAF*	5 (4.5)	2 (6.9)	0.607	3 (4.1)	0	0.313
*KRAS*	42 (38.2)	9 (31.0)	0.477	32 (43.8)	7 (29.2)	0.204
*NRAS*	7 (6.4)	6 (20.7)	**0.029**	3 (4.1)	2 (8.3)	0.417
*HRAS*	2 (1.8)	2 (6.9)	0.146	3 (4.1)	0	0.313
*FBXW7*	6 (5.5)	2 (6.9)	0.767	3 (4.1)	1 (4.2)	0.990
*PTEN*	1 (0.9)	0	0.606	0	0	-
*SMAD4*	4 (3.6)	1 (3.4)	0.961	0	1 (4.2)	0.080
*TGFβ*	1 (0.9)	2 (6.9)	0.110	1 (1.4)	0	0.564
*AKT1*	1 (0.9)	0	0.606	1 (1.4)	0	0.564

Bold: statistically significant.

**Table 4:** Recurrence pattern of colorectal cancer stratified by tumor location.

Metastatic Pattern	All CRC			Right-Sided Colon Cancer			Left-Sided Colon Cancer			Rectal Cancer		
	Early Recurrence *n* = 139 *n* (%)	Late Recurrence *n* = 97 *n* (%)	*p* Value	Early Recurrence *n* = 40 *n* (%)	Late Recurrence *n* = 13 *n* (%)	*p* Value	Early Recurrence *n* = 37 *n* (%)	Late Recurrence *n* = 39 *n* (%)	*p* Value	Early Recurrence *n* = 62 *n* (%)	Late Recurrence *n* = 45 *n* (%)	*p* Value
Local	18 (12.9)	16 (16.5)	0.445	5 (12.5)	0	0.180	2 (5.4)	2 (5.1)	0.957	11 (17.7)	14 (31.1)	0.107
Liver	62 (44.6)	32 (33.0)	**0.048**	16 (40.0)	3 (23.1)	0.269	18 (48.6)	15 (38.5)	0.370	28 (45.2)	14 (31.1)	0.142
Lung	50 (36.0)	45 (46.4)	0.108	12 (30.0)	6 (46.2)	0.285	15 (40.5)	16 (41.0)	0.966	23 (37.1)	23 (51.1)	0.148
Peritoneum	30 (21.6)	14 (14.4)	0.165	16 (40.0)	2 (15.4)	0.104	7 (18.9)	9 (23.1)	0.657	7 (11.3)	3 (6.7)	0.417
Bone	7 (5.0)	7 (7.2)	0.485	2 (5.0)	2 (15.4)	0.218	2 (5.4)	2 (5.1)	0.957	3 (4.8)	3 (6.7)	0.685
Others	6 (4.3)	15 (15.5)	**0.003**	3 (7.5)	2 (15.4)	0.398	2 (5.4)	5 (12.8)	0.264	1 (1.6)	8 (17.8)	**0.003**
Recurrence site			0.482			0.104			0.690			**0.011**
Single site	110 (79.1)	73 (75.3)		28 (70.0)	12 (92.3)		28 (75.7)	31 (79.5)		54 (87.1)	30 (66.7)	
Multiple sites	29 (20.9)	24 (24.7)		12 (30.0)	1 (7.7)		9 (24.3)	8 (20.5)		8 (12.9)	15 (33.3)	

Bold: statistically significant; some patients had more than one metastatic pattern.

**Table 5:** Univariate and multivariate cox regression analysis of overall survival in colorectal cancer with tumor recurrence.

Variables	Univariate Analysis			Multivariate Analysis		
	Hazard Ratio	Confidence Interval	*p* Value	Hazard Ratio	Confidence Interval	*p* Value
Age (year)			**0.007**			**<0.001**
<70	1.00			1.00		
≥70	1.66	1.150–2.410		2.09	1.409–3.088	
Sex			0.937			
Male	1.00					
Female	0.94	0.628–1.399				
Tumor location			0.084			
Right-sided colon	1.00					
Left-sided colon	0.64	0.390–1.047				
Rectum	0.61	0.386–0.962				
Lymphovascular invasion	1.06	0.676–1.662	0.800			
Pathological T category			0.419			
T1	1.00					
T2	0.68	0.087–5.362				
T3	0.67	0.093–4.856				
T4	1.07	0.140–8.110				
Pathological N category			**0.031**			0.163
N0	1.00			1.00		
N1	1.56	1.005–2.406		1.22	0.777–1.900	
N2	1.80	1.133–2.868		1.61	0.986–2.624	
MSI status			0.309			
MSS	1.00					
MSI-high	1.38	0.741–2.576				
Recurrence			**<0.001**			**<0.001**
Early recurrence	1.00			1.00		
Late recurrence	0.45	0.309–0.654		0.41	0.278–0.612	
Recurrence site			**0.001**			**0.012**
Single site	1.00			1.00		
Multiple sites	2.05	1.358–3.104		1.84	1.140–2.969	
Adjuvant chemotherapy	1.26	0.866–1.832	0.228			
Genetic mutation						
*BRAF*	2.20	1.065–4.524	**0.003**	2.94	1.398–6.186	**0.004**
*NRAS*	1.76	1.054–2.934	**0.031**	1.59	0.827–3.044	**0.005**
*HRAS*	1.31	0.481–3.550	0.600			
*TP53*	0.88	0.596–1.298	0.518			
*APC*	0.94	0.613–1.437	0.771			
*PIK3CA*	1.50	0.902–2.495	0.118			
*KRAS*	1.17	0.801–1.694	0.423			
*FBXW7*	1.66	0.840–3.293	0.144			
*PTEN*	4.24	0.583–30.787	0.153			
*SMAD4*	1.39	0.340–5.646	0.650			
*TGFβ*	1.39	0.432–4.465	0.581			
*AKT1*	1.86	0.459–7.570	0.384			

T: tumor; N: node; MSI: microsatellite instability; MSS: microsatellite stable; bold: statistically significant.

**Table 6:** Survival analysis of clinicopathological features and mutation spectrum after curative surgery for colorectal cancer.

Variables	Univariate Analysis				Multiple Testing Correction Logistic Regression		
	Patient Alive *n* = 120 *n* (%)	Patient Died *n* = 116 *n* (%)	Cramer Value	*p* Value	Odds Ratio	Confidence Interval	*p* Value
Age (years)			0.153	**0.019**			**0.016**
<70	69 (57.5)	49 (42.2)			1.000		
≥70	51 (42.5)	67 (57.8)			1.932	1.132–3.297	
Sex			0.069	0.291			
Male	76 (63.3)	81 (69.8)					
Female	44 (36.7)	35 (30.2)					
Tumor location			0.064	0.618			
Right-sided colon	24 (20.0)	29 (25.0)					
Left-sided colon	41 (34.2)	35 (30.2)					
Rectum	55 (45.8)	52 (44.8)					
Tumor differentiation			0.052	0.428			
Well to moderate	112 (93.3)	111 (95.7)					
Poor	8 (6.7)	5 (4.3)					
Lymphovascular invasion	32 (26.7)	24 (20.7)	0.070	0.281			
Recurrence			0.006	0.932			
Early recurrence	71 (59.2)	68 (58.6)					
Late recurrence	49 (40.8)	48 (41.4)					
Adjuvant chemotherapy	72 (60.0)	70 (60.3)	0.004	0.957			
Pathological T category			0.009	0.999			
T1	1 (0.8)	1 (0.9)					
T2	10 (8.3)	10 (8.6)					
T3	94 (78.3)	90 (77.6)					
T4	15 (12.5)	15 (12.9)					
Pathological N category			0.143	0.089			
N0	47 (39.2)	40 (34.5)					
N1	28 (23.3)	42 (36.2)					
N2	45 (37.5)	34 (29.3)					
Pathological TNM stage			0.053	0.717			
I	7 (5.8)	7 (6.0)					
II	40 (33.3)	33 (28.4)					
III	73 (60.8)	76 (65.5)					
MSI status			0.036	0.585			
MSS	111 (92.5)	105 (90.5)					
MSI-high	9 (7.5)	11 (9.5)					
Genetic mutations							
*TP53*	42 (35.0)	38 (32.8)	0.024	0.716			
*APC*	32 (26.7)	28 (24.1)	0.029	0.656			
*PIK3CA*	17 (14.2)	18 (15.5)	0.019	0.770			
*BRAF*	2 (1.7)	8 (6.9)	0.130	**0.046**	4.806	0.983–23.507	0.053
*KRAS*	43 (35.8)	47 (40.5)	0.048	0.459			
*NRAS*	3 (2.5)	15 (12.9)	0.196	**0.003**	6.682	1.854–24.084	**0.004**
*HRAS*	3 (2.5)	4 (3.4)	0.028	0.668			
*FBXW7*	3 (2.5)	9 (7.8)	0.120	0.066			
*PTEN*	0	1 (0.9)	0.066	0.308			
*SMAD4*	4 (3.3)	2 (1.7)	0.051	0.432			
*TGFβ*	1 (0.8)	3 (2.6)	0.068	0.297			
*AKT1*	0	2 (1.7)	0.094	0.149			

MSI: microsatellite instability; MSS: microsatellite stable; TNM: tumor, node, metastasis; bold: statistically significant.

## Data Availability

The data presented in this study are available in the article.

## Abbreviations

CIN	chromosomal instability pathway
CMS	consensus molecular subtype
COSMIC	Catalogue Of Somatic Mutations In Cancer
CRC	colorectal cancer
FAP	familial adenomatous polyposis
FOLFOX	folinic acid, fluorouracil, oxaliplatin
IBD	inflammatory bowel disease
MSI	microsatellite instability
MSS	microsatellite stable
LVI	lymphovascular invasion
OS	overall survival
TASIN-1	truncated *APC* selective inhibitor
TNM	tumor, node, metastasis
